# Co-Stimulation of AGEs and LPS Induces Inflammatory Mediators through PLCγ1/JNK/NF-κB Pathway in MC3T3-E1 Cells

**DOI:** 10.3390/cells12101383

**Published:** 2023-05-13

**Authors:** Natsuko Tanabe, Keiko Tomita, Soichiro Manaka, Risa Ichikawa, Tadahiro Takayama, Takayuki Kawato, Misae Ono, Yuma Masai, Akihisa Utsu, Naoto Suzuki, Shuichi Sato

**Affiliations:** 1 Department of Biochemistry, Nihon University School of Dentistry, 1-8-13 Kanda-Surugadai, Chiyoda-ku, Tokyo 101-8310, Japan; 2 Division of Functional Morphology, Dental Research Center, Nihon University School of Dentistry, 1-8-13 Kanda-Surugadai, Chiyoda-ku, Tokyo 101-8310, Japan; 3 Department of Periodontology, Nihon University School of Dentistry, 1-8-13 Kanda-Surugadai, Chiyoda-ku, Tokyo 101-8310, Japan; 4 Division of Applied Oral Sciences, Nihon University Graduate School of Dentistry, 1-8-13 Kanda-Surugadai, Chiyoda-ku, Tokyo 101-8310, Japan; 5 Department of Oral Health Sciences, Nihon University School of Dentistry, 1-8-13 Kanda-Surugadai, Chiyoda-ku, Tokyo 101-8310, Japan; 6 Division of Oral Structural and Functional Biology, Nihon University Graduate School of Dentistry, 1-8-13 Kanda-Surugadai, Chiyoda-ku, Tokyo 101-8310, Japan; 7 Department of Orthodontics, Nihon University School of Dentistry, 1-8-13 Kanda-Surugadai, Chiyoda-ku, Tokyo 101-8310, Japan

**Keywords:** AGEs, LPS, IL-1α, S100A9, PGE_2_, PLCγ1, JNK, NF-κB

## Abstract

Advanced glycation end-products (AGEs) are increased under hyperglycemia in vivo and are associated with the onset of diabetes. According to previous studies, AGEs exacerbate inflammatory diseases. However, the mechanism by which AGEs aggravate osteoblast inflammation remains unknown. Therefore, the aim of this study was to determine the effects of AGEs on the production of inflammatory mediators in MC3T3-E1 cells and the underlying molecular mechanisms. Co-stimulation with AGEs and lipopolysaccharide (LPS) was found to increase the mRNA and protein levels of cyclooxygenase 2 (COX2), interleukin-1α (IL-1α), S100 calcium-binding protein A9 (S100A9), and the production of prostaglandin E_2_ (PGE_2_) compared to no stimulation (untreated control) or individual stimulation with LPS or AGEs. In contrast, the phospholipase C (PLC) inhibitor, U73122, inhibited these stimulatory effects. Co-stimulation with AGEs and LPS also increased the nuclear translocation of nuclear factor-kappa B (NF-κB) compared to no stimulation (untreated control) or individual stimulation with LPS or AGE. However, this increase was inhibited by U73122. Co-stimulation with AGEs and LPS-induced phosphorylated phospholipase Cγ1 (p-PLCγ1) and phosphorylated c-Jun N-terminal kinase (p-JNK) expression compared to no stimulation or individual stimulation with LPS or AGEs. U73122 inhibited the effects induced by co-stimulation. siPLCγ1 did not increase the expression of p-JNK and the translocation of NF-κB. Overall, co-stimulation with AGEs and LPS may promote inflammation mediators in MC3T3-E1 cells by activating the nuclear translocation of NF-κB via PLCγ1-JNK activation.

## 1. Introduction

Advanced glycation end products (AGEs) are exogenous and endogenous compounds formed from various precursors via different mechanisms in the human body and constitute a chemically diverse group of compounds [1]. AGEs generate non-enzymatic modifications of molecules via the carbonyl groups of reducing sugars and free amine groups of nucleic acids, proteins, or lipids without any biological catalyst produced via further rearrangements, yielding stable, irreversible end products [1].

Type 2 diabetes mellitus is considered one of the most common diseases of the 21st century, expanding with remarkable speed; thus, this condition is expected to affect approximately 693 million adults by 2045 [2]. People with diabetes have a unique susceptibility to complications called diabetic complications. Diabetic complications include end-stage renal failure, various neurological disorders, and arteriosclerosis induced by diabetic micro- and macrovascular complications involving inflammatory disorders due to chronic hyperglycemia [3]. The major cause of diabetic complications is uncontrolled hyperglycemia, which leads to the production of AGEs [2]. AGEs production is moderate under normal physiological conditions and markedly accelerated under continuous hyperglycemic conditions owing to increased glucose availability [2,4].

The initial reactions of AGEs are reversible and depend on serum glucose concentration. AGEs accumulate in every bodily fluid and cause toxic pathogens. Accordingly, AGEs are suggested to be major contributors to diabetic complications, such as Alzheimer’s disease, atherosclerosis, cardiovascular diseases, and cancer [5]. As bioactive substances, AGEs are involved in biological reactions as ligands via their receptors in various cells. The receptor for advanced glycation end products (RAGE) binds and mediates different ligands that damage-associated molecular pattern molecules (DAMPs), including high mobility group 1 (HMGB1), S100s, DNA, and AGEs and is involved in inflammation through various pathways [6,7].

Rheumatoid arthritis (RA) and periodontal diseases are common chronic inflammatory bone diseases. According to the World Health Organization (WHO), more than 23 million worldwide have RA. A previous study revealed that RA leads to the glycation of differential proteins in circulation, resulting in AGEs formation that may activate inflammatory mediators and oxidative stress [7].

Periodontitis is one of the diabetic complications that induces the production of inflammatory cytokines such as interleukin-1 (IL-1), interleukin-6 (IL-6), interleukin-8 (IL-8), interleukin-17 (IL-17), and tumor necrosis factor-α (TNFα) and reduced anti-inflammatory cytokines under hyperglycemia [8]. Periodontal disease is caused by lipopolysaccharide (LPS), an endotoxin derived from Gram-negative bacteria that infects the periodontal tissue and causes inflammation and destruction. LPS induces inflammatory mediators, such as IL-1, IL-6, TNF-α, RANKL, and prostaglandin E_2_ (PGE_2_), which are synthesized by the prostaglandin synthase, cyclooxygenase 2 (COX2) [9,10,11,12]. These inflammatory mediators affect the gingival epithelial cells and alveolar bone, including osteoblasts and osteoclasts [11]. Thus, glycated proteins, including AGEs, may affect the progression of inflammatory bone diseases, such as RA and periodontitis. The accumulation of AGEs involved in diabetes was found to exacerbate periodontitis in a previous study [13]. In addition, the concentration of AGEs in gingival tissues positively correlated with the duration of diabetes [14,15]. However, the signaling pathway by which AGEs are involved in diabetes in hyperglycemia aggravates inflammation of osteoblasts is not clarified.

The aim of this study was to determine the effects of AGEs on the production of inflammatory mediators in MC3T3-E1 cells derived from mouse calvarial cell lines as osteoblast-like cells and the underlying molecular mechanisms.

## 2. Material and Methods

### 2.1. Cell Culture

The MC3T3-E1 mouse calvarial cell line (Riken BioResource Center, Tsukuba, Japan) was used as the osteoblastic cell line. The cells were maintained in α-minimal Essential Medium (α-MEM; FUJIFILM Wako Pure Chemical, Osaka, Japan) containing 10% heat-inactivated fetal bovine serum (HyClone Laboratories, Logan, UT, USA) and 1% Penicillin–Streptomycin–Amphotericin B Suspension (FUJIFILM Wako Pure Chemical) at 37 °C in a humidified atmosphere containing 95% air and 5% CO_2_. The cells were either treated with 100 ng/mL LPS (ligand of TLR4: E. Coli, L4524, Sigma-Aldrich, St. Louis, MO, USA) or left untreated. The medium was replaced every three days.

### 2.2. Preparation of AGEs

AGEs were prepared by incubating 50 mg/mL bovine serum albumin (BSA; Fujifilm Wako Pure Chemical Corporation) and 0.1 M DL-glyceraldehyde (Sigma-Aldrich, St. Louis, MO, USA) under sterile conditions in 0.2 M phosphate-buffered saline (PBS; pH 7.4) containing 5 mM diethylenetriamine pentametric acid (Nacalai Tesque, Kyoto, Japan) at 37 °C for 7 days. Low-molecular-weight reactants and aldehydes were removed using a PD-10 column (GE Healthcare Bio-Sciences AB, Uppsala, Sweden) and dialyzed against PBS [16]. The protein concentration was measured using the Bio-Rad protein assay (Bio-Rad Laboratories, Inc., Hercules, CA, USA). Thereafter, the samples were diluted to 1 mg/mL and kept at −20 °C before use in the experiment.

### 2.3. Real-Time Polymerase Chain Reaction (Real-Time PCR)

The cells were seeded in 6-well plates and cultured for 14 days. Total RNA was isolated on day 14 of culture using the RNeasy Mini Kit (QIAGEN, Valencia, CA, USA). The RNA concentration was measured using NanoDrop 1000 (Thermo Fisher Scientific, Wilmington, DE, USA). Complementary DNA (cDNA) was synthesized from 250 ng of DNase-treated total RNA using PrimeScript™ RT Master Mix (Takara Bio, Shiga, Japan). The resulting cDNA was analyzed via real-time PCR using TB Green^®^ Premix Ex Taq™ II (Takara Bio). The reactions were performed in a total volume of 25 μL, which comprised 12.5 μL of TB Green^®^ Premix Ex Taq™ II, 0.5 μL (10 µM) of each primer (Table 1), 9.5 μL of dH_2_O, and 2 μL (0.25 μg/10 μL) of cDNA. PCR was performed using a Thermal Cycler Dice Real-Time System II (Takara Bio). The following cycling conditions were employed: 35 cycles at 95 °C for 5 s and 60 °C for 20 s. All real-time PCR experiments were performed in triplicate, and the specificity of the amplified product was verified using melting curve analysis. The target mRNA levels were determined for COX2, IL-1α, PLCγ1, and S100A9 and calculated using the ΔCt method. β-actin was used as an internal control.

### 2.4. Enzyme-Linked Immune-Sorbent Assay (ELISA)

After AGEs and/or LPS stimulation, the cells were cultured in serum-free medium for 24 h. The concentrations of IL-1α and prostaglandin E_2_ (PGE_2_) in the culture medium on day 14 were determined using commercially available ELISA kits (IL-1α: Proteintech Group, Inc., Rosemont, IL, USA; PGE_2_: R&D Systems, Minneapolis, MN, USA) according to the manufacturer’s instructions. PGE_2_ and IL-1α concentrations were corrected with the protein concentrations in the culture supernatant, which were measured using a Bio-Rad protein assay (Bio-Rad).

### 2.5. Western Blotting

The cell lysates were prepared using RIPA lysis buffer (ATTO, Tokyo, Japan) containing protease inhibitor cocktail set III (EMD Millipore Corporation, Burlington, CA, USA). First, 60 µg of protein was separated via sodium dodecyl sulfate-polyacrylamide gel electrophoresis (SDS-PAGE) and transferred at polyvinylidene difluoride (PVDF) membranes. The membranes were then blocked using Block-Ace™ (KAC CO., Ltd., Hyogo, Japan) and incubated with COX2 (Cat#12282), SAPK/JNK (JNK; Cat#9252), phosphorylated SAPK/JNK (p-JNK; Cat#4468), phospholipase Cγ1 (PLCγ1; Cat#5690), phosphorylated PLCγ1 (p-PLCγ1; Cat#14008), S100 calcium-binding protein A9 (S100A9; Cat#73425) (Cell Signaling Technology, Danvers, MA, USA), or β-actin (Cat#sc-47778, Santa Cruz Biotechnology, Dallas, TX, USA) containing Western BLoT Immuno Booster solution 1 (Takara bio) at a 1:500 or 1:250 dilution overnight at 4 °C as the first antibody. The membranes were then washed with TBS-Tween and incubated with a mouse IgGκ light chain binding protein conjugated to HRP (Cat#sc-516102), mouse anti-rabbit IgG conjugated to HRP (Cat#sc-2357) (Santa Cruz Biotechnology), goat anti-mouse IgG conjugated to biotin (Cat#AP181B, EMD Millipore Corporation), or mouse anti-rabbit IgG conjugated to biotin (Cat#A27035, Thermo Fisher Scientific) containing Western BLoT Immuno Booster solution 2 (Takara bio) at a 1:2500 dilution for 1 h at room temperature. The primary antibodies against COX2 and S100A9 were incubated with a secondary antibody conjugated to biotin and then treated with peroxidase-leveled streptavidin (LGC SERACARE, Milford, MA, USA). β-actin was used as an internal standard, and the protein bands were detected using Clarity Max™ Western ECL Substrate (Bio-Rad). Immunoreactive proteins were visualized using Amersham™ ImageQuant™ 800 (Cytiva, Tokyo, Japan). The band intensity was quantified using ImageJ software.

### 2.6. Immunofluorescence Localization of NF-κB

Cells were seeded on glass coverslips, fixed with methanol for 15 min at −20 °C, and blocked with Block-Ace™ (KAC Co., Ltd.) for 1 h at room temperature. The cells were then incubated with rabbit monoclonal antibodies against NF-κB p65 (Cat#8242, Cell Signaling Technology) for 1 h at room temperature, followed by an Alexa Fluor 488-conjugated goat-anti-rabbit secondary antibody (Thermo Fisher Scientific, Waltham, MA, USA) for 1 h at room temperature. The signals were detected, and images were acquired using an All-in-One Fluorescence Microscope BZ-X810 (KEYENCE, Osaka, Japan). The images were taken randomly at 5 points per glass coverslip in each sample to calculate the NF-κB p65 nuclear localization proportion. The cells were considered positive for nuclear localization of NF-κB p65 if the fluorescence intensity of their nuclei exceeded that of their cytoplasm. In addition, we calculated the percentage of positive for nuclear localization of NF-κB p65 to total cells in these points for each sample.

### 2.7. Short Interfering (si)RNA

Cells seeded in a 6-well plate were transfected with siRNA (Invitrogen, Carlsbad, CA, USA) against PLCγ1 (siPLCγ1) or a negative control scrambled siRNA (siControl) using Lipofectamine™ RNAiMAX Transfection Reagent (Invitrogen). The transfection complex was prepared as described below. Briefly, 3 µL of 10 µM siRNA was diluted in 150 µL Opti-MEM (Gibco BRL, Rockville, MD, USA) without serum, and 9 µL of the transfection reagent was diluted in 150 µL Opti-MEM. Both mixtures were combined, and 250 µL was added to each well when the cell confluence reached 60–80%. Finally, the cells were incubated for two days. PLCγ1 knockdown was confirmed using real-time PCR and western blotting.

### 2.8. Statistical Analysis

The data represent the results of three or four independent experiments with samples tested in triplicate. Data are expressed as mean and SD. Differences between groups were evaluated using one-way analysis of variance (ANOVA), followed by Tukey’s multiple comparison test or an unpaired *t*-test. Differences were considered statistically significant at *p* < 0.05. Statistical analyses were performed using GraphPad Prism Version 9.5.1 (GraphPad Software, Boston, MA, USA).

## 3. Results

### 3.1. AGEs+LPS Increased the Expression of COX2 and PGE_2_ in MC3T3-E1 Cells

**Figure 1:**
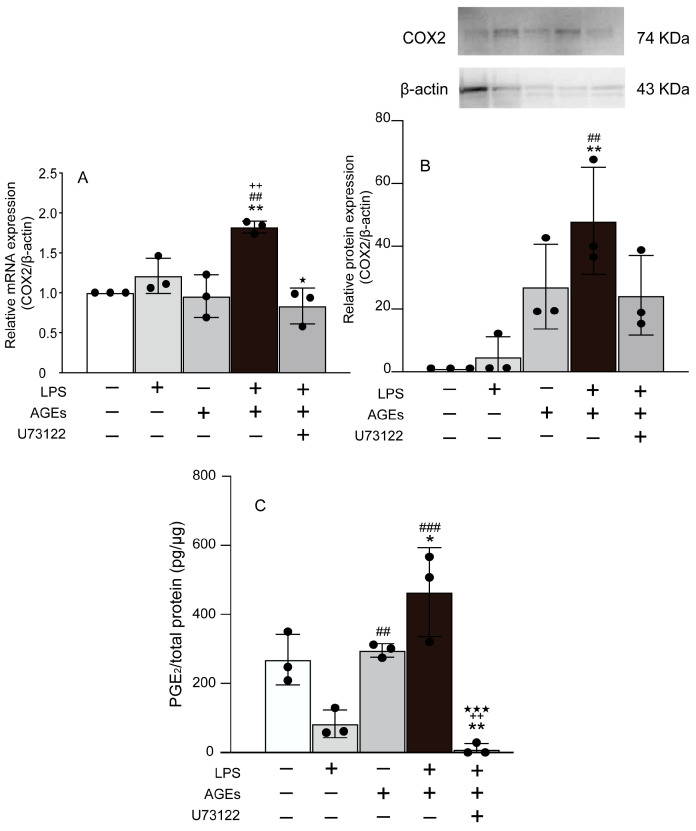
Cells stimulated with or without AGEs (100 μg/mL) and LPS (100 ng/mL), treated with U73122 (10 μM), or not stimulated (untreated control). The mRNA expression of COX2 (**A**) on day 14 of culture based on real-time PCR. The protein expression of COX2 (**B**) on day 14 of culture based on western blotting. The production of PGE_2_ (**C**) on day 14 based on ELISA. Data are expressed as the mean ± SD of three independent experiments performed in triplicate. One-way ANOVA was employed for comparisons between groups while Tukey’s post hoc test was employed for multiple comparisons among all groups. * *p* < 0.05, ** *p* < 0.01 vs. untreated, ^##^ *p* < 0.01, ^###^ *p* < 0.001 vs. LPS, ^++^ *p* < 0.01 vs. AGEs, ^★^ *p* < 0.05, ^★★★^ *p* < 0.001 vs. LPS+AGEs.

We evaluated the co-stimulatory effect of LPS and AGEs on the expression of PGE_2_. AGEs+LPS increased the mRNA and protein levels of COX2 in MC3T3-E1 cells by day 14 of culture compared to the control, LPS alone, and AGEs alone (Figure 1A,B). The PLCγ1 inhibitor, U73122, was found to inhibit the stimulatory effect of LPS+AGE on the mRNA and protein levels of COX2 in cells (Figure 1A,B). Overall, LPS+AGE induced the production of PGE_2_, whereas U73122 inhibited the stimulatory effects of LPS and AGEs (Figure 1C).

### 3.2. AGEs+LPS Increased the Expression of IL-1α, and S100A9 in MC3T3-E1 Cells

**Figure 2:**
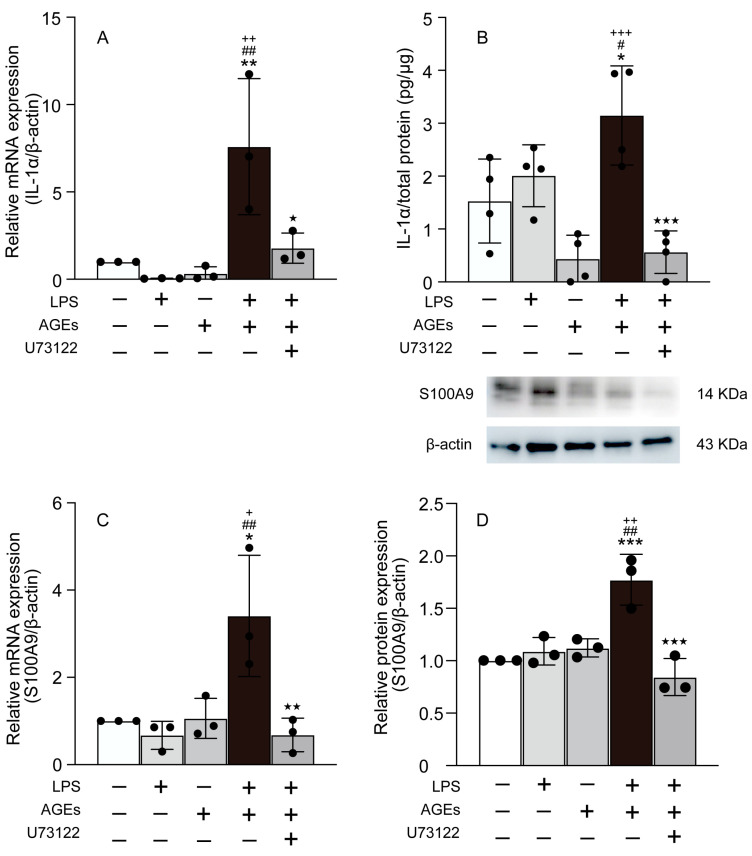
Cells stimulated with or without AGEs (100 μg/mL) and LPS (100 ng/mL), treated with U73122 (10 μM), or not stimulated (untreated control). The mRNA expression of IL-1α (**A**) and S100A9 (**C**) on day 14 of culture based on real-time PCR. The production of IL-1α (**B**) on day 14 of culture based on ELISA. The protein expression of S100A9 (**D**) on day 14 based on western blotting. Data are expressed as the mean ± SD of three or four independent experiments performed in triplicate. One-way ANOVA was employed for comparisons between groups while Tukey’s post hoc test was employed for multiple comparisons among all groups. * *p* < 0.05, ** *p* < 0.01, *** *p* < 0.001 vs. untreated, ^#^ *p* < 0.05, ^##^ *p* < 0.01 vs. LPS, ^+^ *p* < 0.05, ^++^ *p* < 0.01 ^+++^ *p* < 0.001 vs. AGEs, ^★^ *p* < 0.05, ^★★^ *p* < 0.01, ^★★★^ *p* < 0.001 vs. LPS+AGEs.

The effects of LPS+AGEs on IL-1α and S100A9 expression were investigated. On day 14 of culture, LPS+AGEs increased the mRNA and protein expression of IL-1α and S100A9 compared to the control, LPS alone, and AGEs alone (Figure 2). The PLCγ1 inhibitor, U73122, was found to inhibit the stimulatory effect of LPS+AGE on the mRNA and protein levels of IL-1α and S100A9 in cells (Figure 2A–D).

### 3.3. LPS+AGEs Increase NF-kB Nuclear Localization

**Figure 3:**
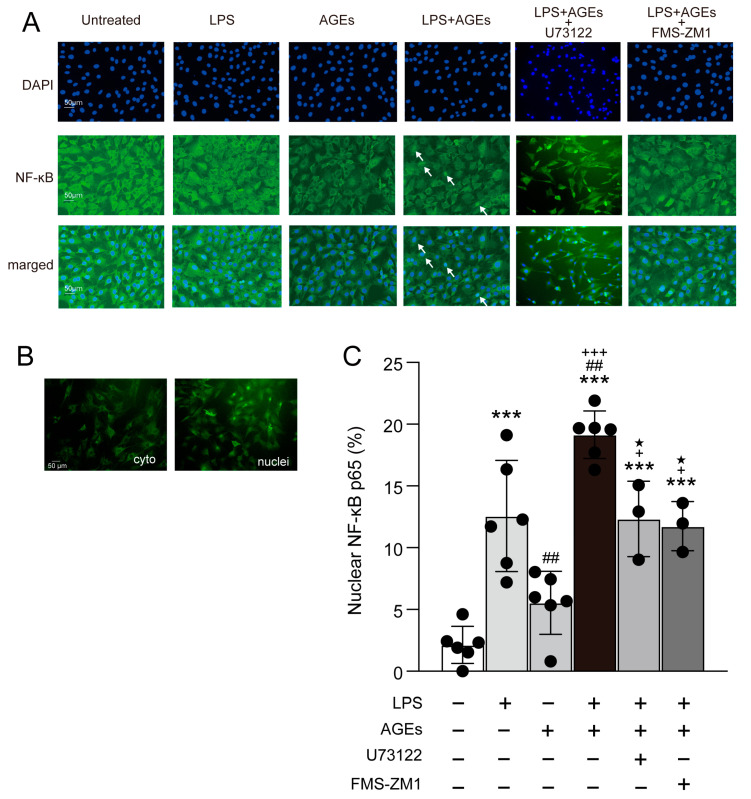
Cells plated on coverslips and stimulated with or without AGEs (100 μg/mL) and LPS (100 ng/mL), treated with U73122 (10 μM) or FPS-ZM1 (40 μM), or not stimulated (untreated control) for 30 min, were fixed with methanol. NF-κB localization was assessed using immunofluorescence. Image of LPS+AGEs-stimulated cells; arrows indicate that NF-κB localized in the nuclei (**A**). Image of a osteoblasts cytoplasmic (cyto) NF-κB localization. Images of osteoblasts showing NF-κB localized in the nuclei (nuclei) (**B**). Bar graph showing the percentage of cells with nuclear localization of NF-κB (**C**). Data are expressed as the mean ± SD of three independent experiments performed in triplicate. One-way ANOVA was employed for comparisons between groups while Tukey’s post hoc test was employed for multiple comparisons among all groups. *** *p* < 0.001 vs. untreated, ^##^ *p* < 0.01 vs. LPS, ^+^ *p* < 0.05, ^+++^ *p* < 0.001 vs. AGEs, ^★^ *p* < 0.05 vs. LPS+AGEs.

To investigate the signaling pathway by which LPS+AGEs increase the levels of inflammatory mediators, we detected the nuclear localization of NF-κB by immunohistochemistry after co-stimulation with LPS and AGEs containing U73122 for 30 min. LPS+AGE induced the nuclear accumulation of NF-κB (Figure 3A). However, this effect was inhibited by U73122 (Figure 3). We proceeded to detect the nuclear localization of NF-κB after co-stimulation with AGEs and LPS and the RAGE inhibitor, FPS-ZM1, at 30 min. Notably, FPS-ZM1 inhibited the stimulatory effects of AGEs and LPS on the nuclear accumulation of NF-κB (Figure 3).

### 3.4. LPS+AGEs Increased p-PLCγ1 and p-JNK46 in MC3T3-E1 Cells

**Figure 4:**
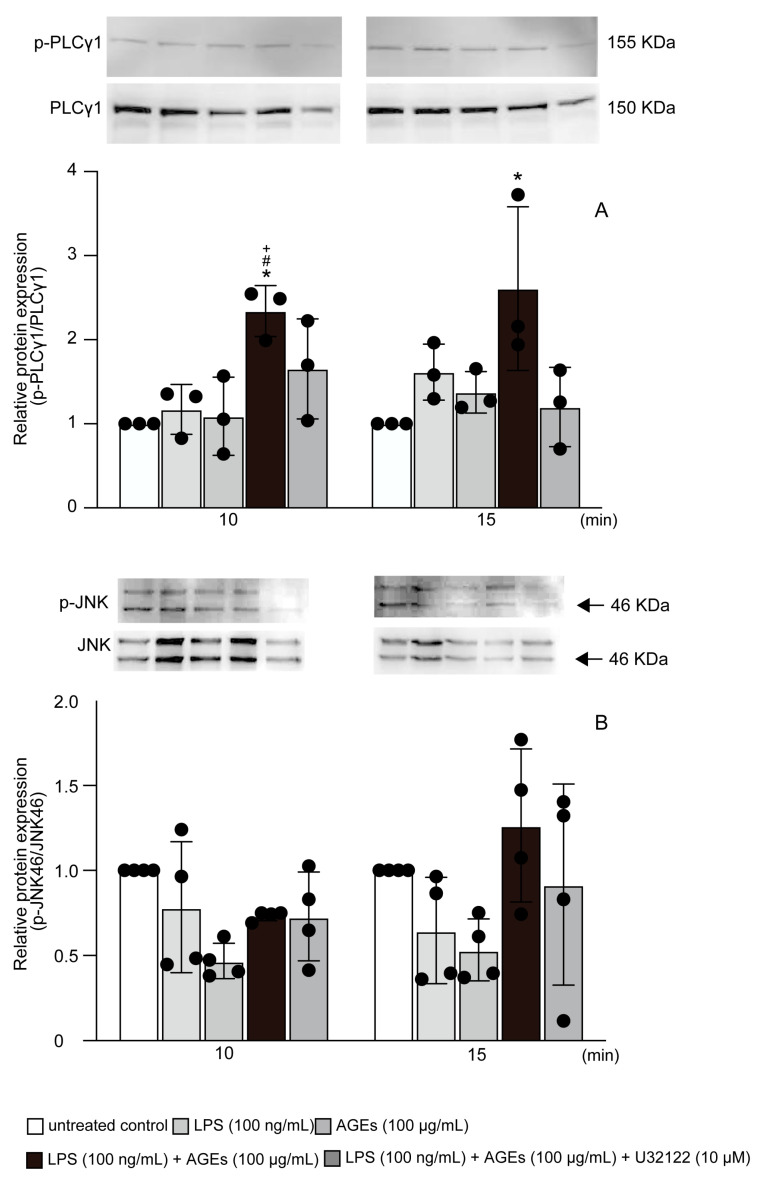
Cells stimulated with or without AGEs (100 μg/mL) and LPS (100 ng/mL), and treated with U73122 (10 μM), or not stimulated (untreated control) for 10 and 15 min. The protein expression of p-PLCγ1 (**A**) and p-JNK (**B**) based on western blotting. Data are expressed as the mean ± SD of three or four independent experiments performed in triplicate. One-way ANOVA was employed for comparisons between groups while Tukey’s post hoc test was employed for multiple comparisons among all groups. * *p* < 0.05 vs. untreated, ^#^ *p* < 0.05 vs. LPS, ^+^ *p* < 0.05 vs. AGEs.

To investigate the downstream signaling pathway involved in the co-stimulatory effect of AGEs and LPS on inflammation, we detected the protein expression of p-PLCγ1 and p-JNK using western blotting after cells were stimulated with LPS and AGEs and treated with U73122. LPS+AGEs increased p-PLCγ1 expression at 10 and 15 min and p-JNK expression at 15 min (Figure 4A,B); these increases were inhibited by U73122 (Figure 4).

### 3.5. AGEs+LPS Increased the Nuclear Translocation of NF-κB Nuclear Localization through the PLCγ1-JNK Pathway

**Figure 5:**
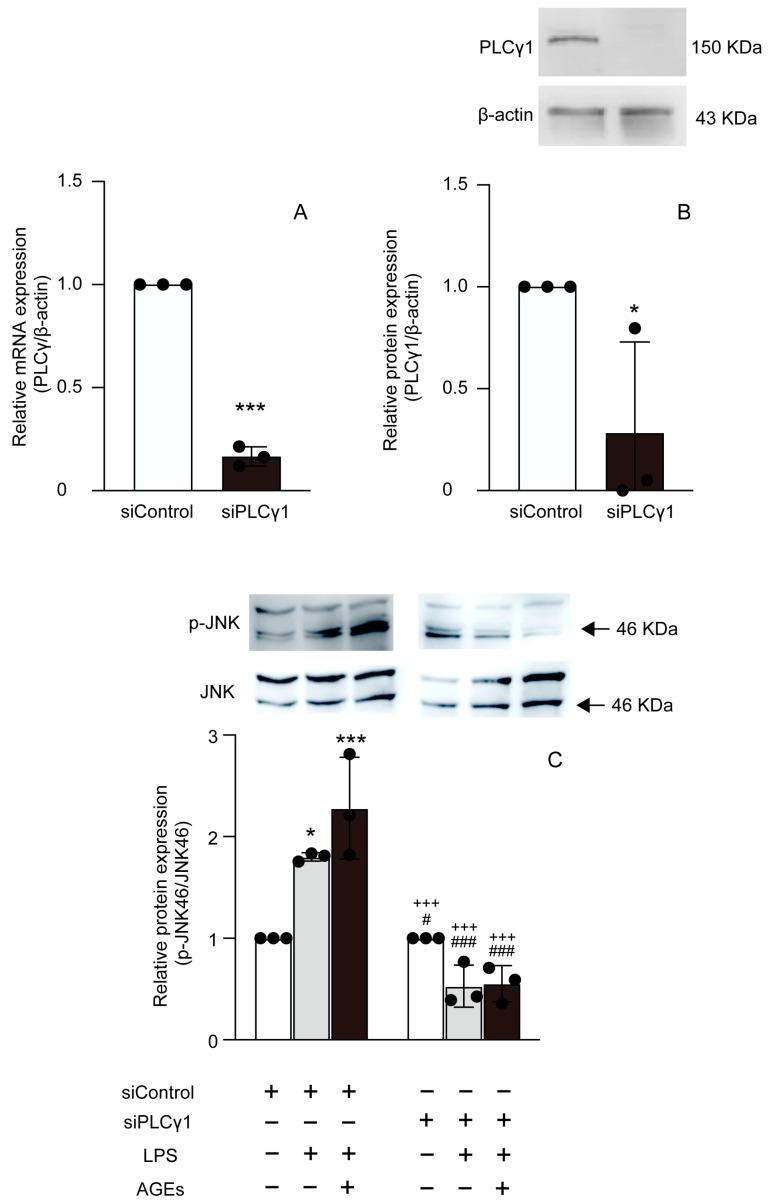
Cells were transfected with PLCγ1-specific siRNA (siPLCγ1) or control scrambled siRNA (siControl). The expression of PLCγ1 mRNA and protein based on real-time PCR (**A**) and western blotting (**B**). Cells stimulated with AGEs (100 μg/mL) and LPS (100 ng/mL) or not stimulated (untreated control) for 10 and 15 min. The protein expression of p-JNK (**C**) based on western blotting. Data are expressed as the mean ± SD of three independent experiments performed in triplicate. Unpaired *t*-test was employed for comparisons between two groups. * *p* < 0.05, *** *p* < 0.001 vs. siControl untreated, ^#^ *p* < 0.05, ^###^ *p* < 0.001 vs. siControl LPS, ^+++^ *p* < 0.001 vs. siControl LPS+AGEs.

**Figure 6:**
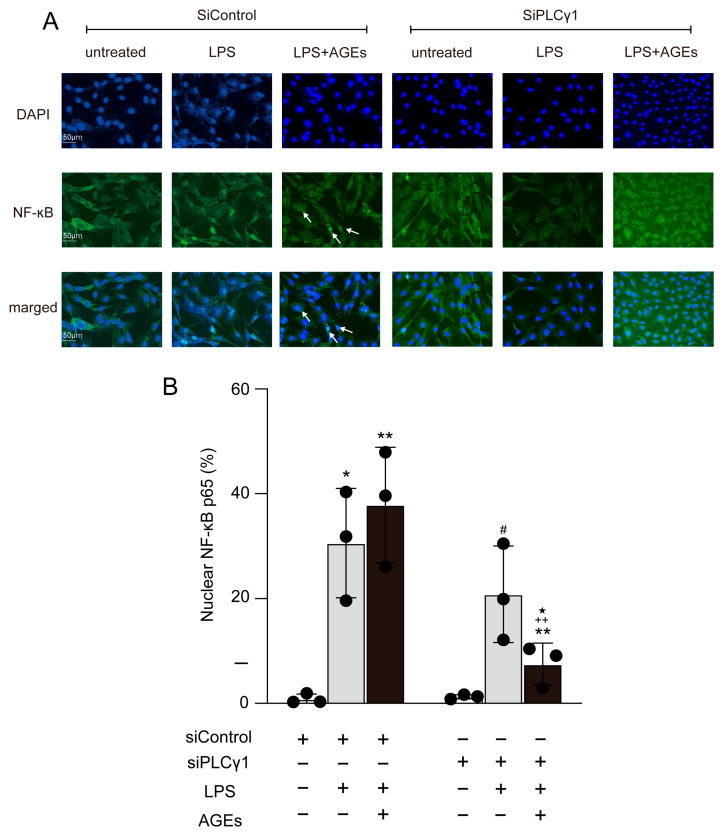
Cells were transfected with PLCγ1-specific siRNA (siPLCγ1) or control scrambled siRNA (siControl) and either stimulated or not stimulated with AGEs (100 μg/mL) and LPS (10 μg/mL). After 30 min of AGEs and LPS co-stimulation, the cells were fixed with methanol. NF-κB localization was assessed using immunofluorescence. Image of LPS+AGEs-stimulated cells; arrows indicate that NF-κB localized in the nuclei (**A**). Bar graph showing the percentage of cells exhibiting nuclear localization of NF-κB (**B**). Data are expressed as the mean ± SD of three independent experiments performed in triplicate. One-way ANOVA was employed for comparisons between groups while Tukey’s post hoc test was employed for multiple comparisons among all groups. * *p* < 0.05, ** *p* < 0.01 vs. siControl untreated, ^#^ *p* < 0.05 vs. siControl LPS, ^++^ *p* < 0.01 vs. siControl LPS+AGEs, ^★^ *p* < 0.05 vs. siPLCγ1 LPS.

We proceeded to determine the effect of PLCγ1 on the nuclear localization of NF-κB induced by LPS and AGEs. The cells were transfected with siPLCγ1, and control scrambled siRNA (siControl). siPLCγ1 cells displayed reduced mRNA and protein expression of PLCγ1 compared to siControl cells (Figure 5A,B). Furthermore, siPLCγ1 abolished the stimulatory effects of LPS and AGEs on JNK phosphorylation in cells (Figure 5C). Cells transfected with siPLCγ1 and co-stimulated with AGEs and LPS showed decreased nuclear translocation of NF-κB compared to those transfected with siControl and stimulated with LPS or co-stimulated with AGEs and LPS (Figure 6).

## 4. Discussion

Inflammation is a physiological response to injury or stimulation caused by bacterial infections, chemical agents, or heat [17]. LPS is the main component of Gram-negative bacterial membranes and is used as a typical stimulator of the inflammatory response in various cell experiments [18]. LPS binds to Toll-like receptor (TLR) 4, TLR2, and RAGE-involved inflammation, leading to inflammatory cytokines/chemokines [18,19]. Notably, LPS induces the production of the inflammatory mediators IL-1, IL-6, PGE_2_, and TNFα. LPS also affects periodontal soft tissues and alveolar bone, which contains osteoblasts and osteoclasts [11]. Inflammatory bone diseases, such as RA and periodontitis, have been implicated in AGEs accumulation [4,13,14,15]. Therefore, in the present study, LPS was used to induce inflammation in osteoblasts. In addition, the effects of AGEs on the expression of inflammatory mediators and their underlying molecular mechanisms were elucidated.

PGE_2_ is an eicosanoid lipid mediator synthesized from arachidonic acid by COX enzymes. PGE_2_ is produced by nearly all cells but is not stored in cells [20,21,22,23]. PGE_2_ responds to cell-specific stimuli, trauma, and signaling molecules and increases RANKL and IL-6 production. In contrast, the COX2 inhibitor, NS398, inhibits RANKL and IL-6 production in MC3T3-E1 cells [24]. These findings indicate that COX2 synthesizes PGE_2_ and induces IL-6 and RANKL expression in osteoblasts.

IL-1α is a family cytokine member that triggers innate inflammation via the IL-1 family of receptors and functions as DAMPs [25]. The interleukin-1 family members are linked to inflammation. However, numerous biological properties of the IL-1 family are nonspecific. The IL-1 family member, IL-1α, functions as a DAMP. Although the inflammatory properties of the IL-1 family dominate innate immunity, IL-1 family members can also play a role in acquired immunity. Hence, the induction of COX2 and various cytokines and chemokines, increased expression of adhesion molecules, and synthesis of nitric oxide in fundamental inflammatory responses are indistinguishable responses to both IL-1 and TLR ligands [25].

The S100 family consists of 21 structurally related members with diverse functions and expression patterns [8]. S100s are small proteins characterized by two EF-hand motifs that play critical roles inside the cell as calcium sensors, cell growth and differentiation modulators, and organizers of the actin cytoskeleton [8]. S100s are also released by multiple cell types in the inflammatory state, where they likely act as cytokines and DAMPs. RAGE binds to and mediates the effects of other S100s, including S100A9 [8]. S100A8 (Calgranulin A or MRP8), and S100A9 (Calgranulin B or MRP14), known as calgranulins, are expressed and secreted by myeloid cell types from the extracellular space as heterodimers [8]. A previous study revealed that AGEs and LPS increased S100A8/A9 expression via NF-κB in human gingival epithelial cells [26]. S100A9 induced the gene expression of IL-1 via NF-κB in human nucleus pulposus cells [27]. Thus, we hypothesized that co-stimulation with AGEs and LPS would affect the expression of inflammatory mediators, such as IL-1α, PGE_2_, and S100A9, in MC3T3-E1 cells. Consequently, co-stimulation with AGEs and LPS induced the mRNA and protein expression of IL-1α, COX2 (a PGE_2_ synthase), S100A9, and the production of PGE_2_ (Figure 1 and Figure 2). In contrast, the PLC inhibitor, U73122, abolished the co-stimulatory effects of AGEs and LPS (Figure 1 and Figure 2). In the present study, the expression of S100A8, which forms a heterodimer with S100A9, was not observed. Therefore, the implications of S100A8/A9 induced by co-stimulation with AGEs and LPS on the expression of inflammatory mediators and the molecular mechanisms will be elucidated in future studies.

NF-κB is one of the most important regulators of proinflammatory gene expression and regulates the synthesis of cytokines, TNF-α, IL-1β, IL-6, and IL-8, similar to the expression of COX2 [28]. NF-κB has five dimers in mammals: p65 (RelA), RelB, c-Rel, p50 (NF-κB1), and p52 (NF-κB2). NF-κB p50 and p65 are composed of heterodimers and have an inhibitory protein, IκB, in the cytoplasm [29,30]. Activation of NF-κB kinase leads to IKKα and IKKβ, which trigger phosphorylated serine residues in IκB for the degradation and ubiquitination of IκB in the proteasome [29]. The degradation and ubiquitination of IκB induce the translocation of NF-κB to the nucleus to activate gene transcription [29,31]. A previous study revealed that epidermal growth factor (EGF) stimulation activated NF-κB via PLCγ1 in colorectal cancer cells [32]. LPS was found to increase the phosphorylation of PLCγ1. Further, the activation of NF-κB caused osteoclastogenesis in RAW264.7 and increased the serum level of TNF-α in vivo [33]. Thus, we proceeded to determine the effects of co-stimulation with AGEs and LPS on the nuclear translocation of NF-κB and involved PLCγ1. Based on our previous studies, the nuclear translocation of NF-κB in MC3T3-E1 cells was most affected at 5 min after low-intensity pulsed ultrasonic stimulation [12]. Further, 30 min of stimulation was found to have the greatest effect relative to that of the controls stimulated with LPS or AGEs in this study. The difference in time between these reports was due to the different types of LPS, which may have caused different responses. However, both reports suggest that the nuclear translocation of NF-κB induced a response within a short period. We hypothesized that LPS and AGEs responses molecule NF-κB in a short period. Co-stimulation with LPS and AGEs was continued for 14 days. Co-stimulation with LPS and AGEs was found to enhance NF-κB nuclear translocation at 30 min and increase PGE_2_, IL-1α, and S100A9 production. However, treatment with U73122 inhibited the co-stimulatory effects of LPS and AGEs at day 14 (Figure 3). Collectively, these results suggest that a repeat of NF-κB nuclear translocation in a short period induces inflammatory mediators on day 14 in cells co-stimulated with LPS and AGEs, and U73122 suppresses the co-stimulation of LPS- and AGE-induced inflammatory mediator production by increasing NF-κB nuclear translocation. The RAGE inhibitor, FPS-ZM1, inhibited the co-stimulatory effect of AGEs and LPS on the nuclear translocation of NF-κB (Figure 3). AGEs induced NF-κB activation via RAGE in HUVEC cells and GLUTag cells [34,35]. These results suggest that IL-1α, PGE_2_, and S100A9 are induced by co-stimulation with AGEs and LPS via PLCγ1-NF-κB. Next, we focused on the downstream pathways of RAGE and TLR4 activated by co-stimulation with AGEs and LPS. LPS induced IL-1β and COX2 via TLR4-JNK-NF-κB in mouse epithelial cells [36]. According to a previous study, HMGB1 and DAMP increased the protein expression of p-NF-κB and p-JNK. In contrast, RAGE antibody-treated cells inhibited the stimulatory effects of HMGB1 on normal human bronchial epithelial cells [37]. Thus, RAGE ligands, including AGEs and S100A9, are activated via the JNK-NF-κB pathway. We examined the effects of co-stimulation with AGEs and LPS on the protein expression of p-PLCγ1 and p-JNK at 10 and 15 min. Co-stimulation with AGEs and LPS increased p-PLCγ1 protein expression at 10 and 15 min and p-JNK protein expression at 15 min; however, U73122 blocked these increases (Figure 4). Furthermore, we examined the implication of PLCγ1 on the JNK-NF-κB pathway induced by co-stimulation with AGEs and LPS. siPLCγ1 did not increase p-JNK protein expression and NF-κB nuclear translocation in cells co-stimulated with AGEs and LPS (Figure 5 and Figure 6). However, as shown in Figure 3, the percentage of nuclear translocation of NF-κB in cells treated with the PLC inhibitor, U73122, and the RAGE inhibitor, FMS-ZM1, was similar to that in cells treated with LPS. Furthermore, compared to cells transfected with siControl, the nuclear translocation of NF-κB was not inhibited in cells transfected with siPLCγ1 and stimulated with LPS. According to some previous studies, LPS acts NF-κB activation via MyD88-ERK1/2 [12,38,39,40]. Thus, LPS may also affect the ERK1/2-NF-κB pathway.

## 5. Conclusions

Co-stimulation with AGEs and LPS was found to increase the production of PGE_2_, IL-1α, and S100A9 via the PLCγ1/JNK/NF-κB pathway. Thus, p-PLCγ1 is important for the increase in inflammatory mediators induced by the co-stimulation of LPS and AGEs. These findings indicate that AGEs may exacerbate LPS-induced inflammation. Further, the molecular mechanism underlying the co-stimulatory effect of AGEs and LPS on inflammation suggests a potential treatment approach for inflammatory bone diseases, including rheumatoid osteoarthritis and periodontitis, under diabetic conditions.

## Figures and Tables

**Table 1:** PCR primers used in the experiments.

Target	Primers	GenBank Acc.
IL-1α	5′-TGGTTAAATGACCTGCAACAGGAA-3′ 5′-AGGTCGGTCTCACTACCTTGTGATG-3′	NM_010554.4
COX2	5′-GCCAGGCTGAACTTCGAAACA-3′ 5′-GCTCACGAGGCCACTGATACCTA-3′	NM_011198.5
S100A9	5′-ACCACCATCATCGACACCTTC-3′ 5′-AAAGGTTGCCAACTGTGCTTC-3′	NM_009114.3
PLCγ	5′-CGTCAACGTGGAGGACAAGA-3′ 5′-ATCACCGAAGGACAGCTTGG-3′	NM_172285.2
β-actin	5′-CATCCGTAAAGACCTCTATGCCAAC-3′ 5′-ATGGAGCCACCGATCCACA-3′	NM_007393.5

## Data Availability

The data that support the findings of this study are available from the corresponding author upon reasonable request.

## Abbreviations

AGEs	advanced glycation end-products
RAGE	receptor for advanced glycation end-products
DAMPs	damage-associated molecular pattern molecules
HMGB1	high mobility group 1 or amphoterin
RA	rheumatoid arthritis
IL-1	interleukin-1
IL-6	interleukin-6
IL-8	interleukin-8
IL-17	interleukin-17
TNFα	tumor necrosis factor-α
RANKL	receptor activator of nuclear factor-kappa B ligand
LPS	lipopolysaccharide
RA	rheumatoid arthritis
PGE_2_	prostaglandin E_2_
COX2	cyclooxygenase 2
ELISA	enzyme-linked immuno-sorbent assay
PLC	phospholipase C
p-PLCγ1	phosphorylated Phospholipase Cγ1
NF-κB	nuclear factor-kappa B
JNK:	c-Jun N-terminal kinase
p-JNK	phosphorylated c-Jun N-terminal kinase
S100A9	S100 calcium binding protein A9
TLR	toll-like receptor
EGF	epidermal growth factor
